# Isolation and Mechanistic Investigation of the Efficient Zearalenone-Removing Strain *Bacillus licheniformis* YJ25

**DOI:** 10.3390/toxins17060263

**Published:** 2025-05-23

**Authors:** Yuting Wu, Feina Wu, Pan Zhao, Yan Gao, Mengyao Li, Mengjiao Luo, Qian Zhou, Siyuan Zhou, Xinhui Li, Yaling Hong, Yang Wu, Zhaorong Zhou, Yang Liu, Yandong Xia, Lijun Zou, Jia Yin

**Affiliations:** 1 Hunan Provincial Key Laboratory of Animal Intestinal Function and Regulation, Hunan International Joint Laboratory of Animal Intestinal Ecology and Health, College of Life Sciences, Hunan Normal University, Changsha 410081, China; wuyuting12025@163.com (Y.W.); wfn13975901301@163.com (F.W.); zp177113115@163.com (P.Z.); gaoyan121724@163.com (Y.G.); lmyao0313@163.com (M.L.); mengjiao_luo@163.com (M.L.); zq66880908@163.com (Q.Z.); sy_zhou178@163.com (S.Z.); jg19918490631@163.com (X.L.); wazzihhhy@163.com (Y.H.); wuyang6810@139.com (Y.W.); zh13617486093@163.com (Z.Z.); l15083794939@163.com (Y.L.); 2 College of Life and Environmental Sciences, Central South University of Forestry and Technology, Changsha 410004, China; 3 Laboratory of Basic Biology, Hunan First Normal University, Changsha 410205, China

**Keywords:** Zearalenone, *Bacillus licheniformis*, peptidoglycan, teichoic acid

## Abstract

Zearalenone (ZEN), a non-steroidal estrogenic mycotoxin produced by *Fusarium graminearum* species, poses a significant threat to both human food safety and animal feed quality. In this study, we isolated a strain, designated as *Bacillus licheniformis* YJ25, from a contaminated moldy corn sample, demonstrating substantial effectiveness in removing ZEN. Our findings revealed that YJ25’s ZEN removal occurs primarily through cell wall adsorption, with enzymatic degradation representing a potential mechanism. In practical applications, enzymatic degradation may yield metabolites with heightened toxicity. However, liquid chromatography–mass spectrometry (LC–MS) analysis revealed that ZEN was not converted into α-/β-zearalenol (α-/β-ZEL) or α-/β-zearalanol (α-/β-ZAL) by YJ25, substantiating the safety profile of YJ25 in the removal of ZEN. Our mechanistic investigations revealed that the cell wall components peptidoglycan and teichoic acid serve as the primary binding sites for ZEN adsorption. Fourier-transform infrared spectroscopy (FTIR) analysis identified O-H, C-H, C=O, and C-O as the principal functional groups participating in the cell wall adsorption process. These investigations establish a scientific foundation for the prospective application of this strain as an efficient biological detoxification agent in food and feed safety management systems.

## 1. Introduction

Aflatoxins (AF), fumonisin (FUM), ochratoxin A (OTA), trichothecenes such as deoxynivalenol (DON), and ZEN represent the most prevalent mycotoxins often detected in animal feed [1]. Among these, ZEN, a nonsteroidal estrogenic mycotoxin, is ubiquitously present in both human dietary sources and animal feedstuffs [2]. This toxic secondary metabolite is primarily produced by *Fusarium graminearum*, initially isolated from maize inoculated with *Fusarium* [3]. ZEN contamination is commonly observed in staple food crops, including maize, wheat, and barley, as well as in various herbal medicinal products [4,5]. Studies have demonstrated the presence of ZEN along with its phase II sulfate and glucoside metabolites in various food and feed samples. Following ingestion, these conjugated forms of ZEN can undergo hydrolysis mediated by intestinal microbiota, resulting in the release of free ZEN [6]. The mycotoxin and its reduced derivatives function as competitive substrates for key steroidogenic enzymes, specifically 3α-hydroxysteroid dehydrogenase and 3β-hydroxysteroid dehydrogenase, thereby potentially disrupting steroid biosynthesis pathways [7].

Regulatory agencies have established rigorous control measures for ZEN contamination. The European Union (EU) legislation sets the maximum permissible levels of ZEN at 2 ppm in cereals and cereal products (excluding corn by-products) and 3 ppm in corn by-products, reflecting the international concern for controlling this mycotoxin’s presence in feed. Similarly, China’s GB13078-2017 Feed Hygiene Standard enforces more rigorous thresholds, limiting ZEN concentrations to 0.5 mg/kg in corn and its processed products (excluding corn hulls, sprayed corn hulls, and dried corn syrup) [8,9]. Given these regulatory constraints, the implementation of comprehensive strategies to mitigate *Fusarium graminearum* infection and subsequent ZEN biosynthesis is crucial.

The mitigation of ZEN contamination can be achieved through two primary strategies: Inhibiting the growth of *Fusarium graminearum* and developing effective detoxification methods for contaminated grains. Detoxification methods are broadly categorized into physical, chemical, and biological approaches. Physical methods include radiation treatment, heat treatment, and adsorption techniques [10,11]. Chemical methods have also demonstrated detoxification potential [12]. However, physical methods often fall short of completely eliminating ZEN, while chemical methods are hindered by high risks, low efficiency, and the potential generation of toxic byproducts, making them unsuitable for large-scale applications [13]. In contrast, biological detoxification methods offer several advantages, including scalability, preservation of nutrient content in feed, high specificity, efficiency, eco-friendliness, and the avoidance of secondary contamination. These attributes make biological detoxification particularly effective for ZEN degradation. Consequently, biological detoxification is regarded as one of the most promising strategies for future ZEN mitigation [14].

Biological detoxification primarily operates through two distinct mechanisms. The first mechanism involves the adsorption of toxins by microorganisms via their external structures, particularly cell wall components. The second mechanism entails the secretion of specific enzymes by microorganisms, which catalyze the conversion of toxins into less toxic or non-toxic compounds. The enzymatic approach is often limited by its inherent sensitivity to pH variations, rendering it ineffective under extreme acidic or alkaline conditions. Furthermore, bioconversion does not inherently imply detoxification, since the metabolites produced may maintain or even display increased toxicity. A notable example is the enzymatic degradation of ZEN, which produces α-zearalanol and β-zearalanol—metabolites that remain estrogenic analogs with potential biological activity [15,16]. In contrast, microbial cell wall adsorption removes toxins through direct physical binding, eliminating potential risks associated with toxic metabolites and ensuring significant safety. Although enzymatic degradation is more efficient, further research on ZEN removal via cell wall adsorption remains crucial. As research on microbial-based ZEN mitigation has gained increasing attention, studies reporting the detoxification of mycotoxins through adsorption have also grown significantly.

Microorganisms, such as bacteria and yeasts, are known to adsorb mycotoxins through various cell wall components, including proteins, carbohydrates, and peptidoglycans. For instance, El-Nezami et al. demonstrated that the *Lactobacillus rhamnosus* strain binds mycotoxins via interactions with polysaccharides in its cell wall [17]. Niderkorn et al. demonstrated that exposed peptidoglycan components in the cell wall of lactic acid bacteria effectively adsorb fumonisin B_1_ and B_2_ [18]. Similarly, He et al. identified teichoic acid and peptidoglycan molecules on the cell wall of *Lactobacillus* strain 12-6 as the primary binding sites for ZEN adsorption [19]. Furthermore, Zoghi et al. revealed that both yeast and bacterial cells can adsorb mycotoxins through specific proteins and carbohydrate components present in their cell walls [20]. Additionally, Swamy et al. demonstrated that glucomannan in yeast cell walls exhibits significant mycotoxin adsorption capacity [21]. In research focusing on lactic acid bacteria, Wang et al. revealed that lipoteichoic acids and envelope proteins play crucial roles in maintaining cell wall surface hydrophobicity, while functional groups (C-O, O-H, and N-H) associated with specific proteins and carbohydrate components also contribute to the adsorption process [22]. However, research on the identification and mechanistic characterization of safe and effective ZEN-adsorbing strains remains limited.

Previous research identified *Bacillus licheniformis* CK1, a soil-derived strain, as capable of both removing and detoxifying ZEN in feed [23,24]. Building upon these findings, Tsui-Chun Hsu et al. systematically characterized the probiotic properties of CK1 and evaluated its ZEN removal efficacy. To evaluate the stability of mycotoxin-adsorbent complexes in the animal digestive tract, Hsu et al. subjected the CK1-ZEN complex to multiple washing procedures. Remarkably, after five washing cycles, more than 30% of ZEN was retained within the hydrophobic pockets of CK1 cells via physical adsorption processes [25]. These results demonstrate microbial-specific variation in mycotoxin-binding stability, with CK1 exhibiting particularly robust ZEN adsorption ability.

Furthermore, the study examined environmental influences on the ZEN removal efficacy of CK1. Heat treatment and acid exposure had no significant impact on CK1’s detoxification capacity, further affirming its robust functionality. Collectively, these findings underscore both the feasibility and superior performance of microbial adsorption as a detoxification strategy.

Moreover, the *Bacillus licheniformis* strains studied by Sorokulova et al. were all non-hemolytic and did not produce hemolysin BL (Hbl) or non-hemolytic enterotoxin (Nhe) enterotoxins [26]. Chronic toxicity studies conducted on mice, rabbits, and swine showed no evidence of toxicity or histological alterations in organs and tissues. This indicates that some *Bacillus licheniformis* strains can be considered non-pathogenic and safe for animal consumption. Additionally, according to the Catalogue of Feed Additives Species issued by the Ministry of Agriculture, *Bacillus licheniformis* is listed among the microbial feed additives used for farm animals, further demonstrating its potential feasibility for use as a feed additive. Currently, the identification of safe and non-toxic microbial strains capable of removing ZEN, as well as the elucidation of their specific removal mechanisms, remain key areas of ongoing research.

In this study, we isolated a strain, designated as YJ25, demonstrating efficient ZEN removal ability, and identified it both genotypically and phenotypically. Through comparative analysis of the 16S rDNA gene sequence, YJ25 was identified as *Bacillus licheniformis*. Meanwhile, whole genome analysis revealed the general genomic features of YJ25, including protein-coding genes, genome size, and other genetic characteristics. Additionally, we systematically evaluated the temporal dynamics of ZEN removal by YJ25 and elucidated the mechanism of ZEN removal by YJ25 through localization and analysis of the adsorption sites on the cell wall. Based on the FTIR and LC–MS, we further confirmed that YJ25 does not generate α-/β-ZEL and α-/β-ZAL during this process and investigated the specific mechanisms underlying YJ25’s adsorption of ZEN.

## 2. Results and Discussion

### 2.1. Isolation and Characterization of ZEN-Removing Bacteria

#### 2.1.1. Isolation of ZEN-Removal Strains

**Figure 1:**
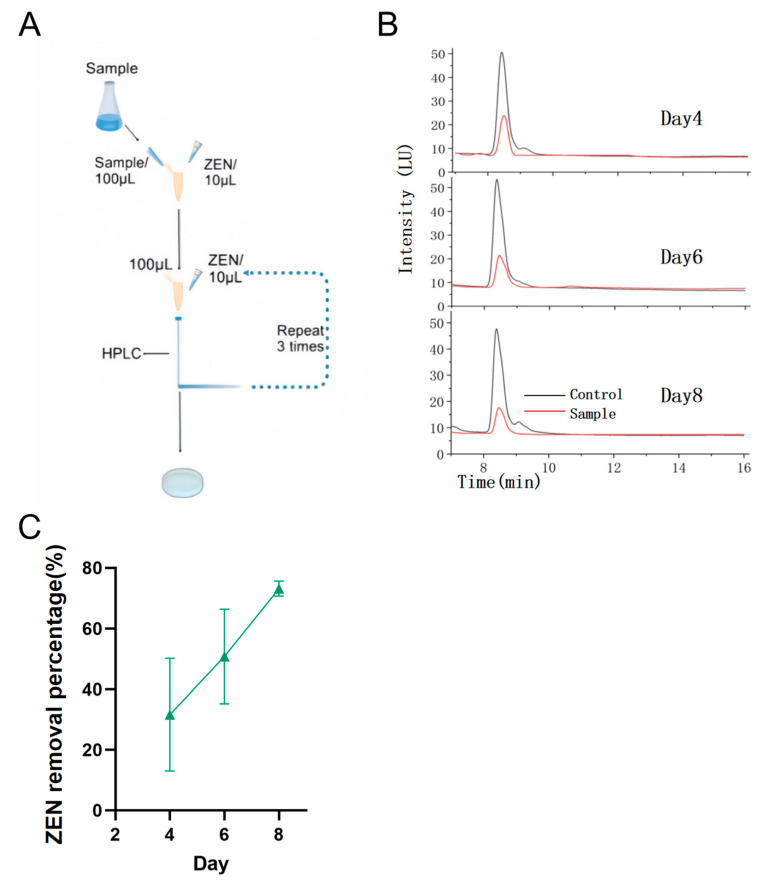
Effects of multiple enrichment cultures on ZEN removal by the sample. (**A**) The operation overview of enrichment culture. (**B**) HPLC profiles of ZEN removal by the sample following enrichment culture. The initial ZEN concentration was 10 μg/mL. (**C**) The removal percentage curve of ZEN from the first to the fourth enrichment cultures.

The moldy corn sample was purchased from Tang Ren Shen Group Co., Ltd. (Zhuzhou, China), 2024, and the procedure of enrichment cultures is illustrated in Figure 1A.

The ZEN removal efficiency was quantified using high-performance liquid chromatography (HPLC), reflecting the ZEN-detoxifying capacity of the sample or strains: Figure 1B,C clearly shows a gradual increase in the removal percentage of ZEN from a moldy corn sample throughout the enrichment culture process. This observation indicated a consistent accumulation of microbial populations with high ZEN-removing efficiency within the culture medium. Following four cycles of enrichment culture, the corn sample exhibited a ZEN removal efficiency of 74.2%, confirming the presence of highly effective ZEN-removing microbial strains in the corn sample. Through systematic screening, we isolated two strains demonstrating stable growth. Using standard streak plate methodology, these isolates were purified and quantitatively evaluated for their ZEN removal capacity. The strain showing superior detoxification efficiency, designated as YJ25, was subsequently selected for further characterization. This microbial strain has been officially deposited in the China General Microbiological Culture Collection Center in Beijing (CGMCC No. 33578).

#### 2.1.2. Identification of YJ25

**Figure 2:**
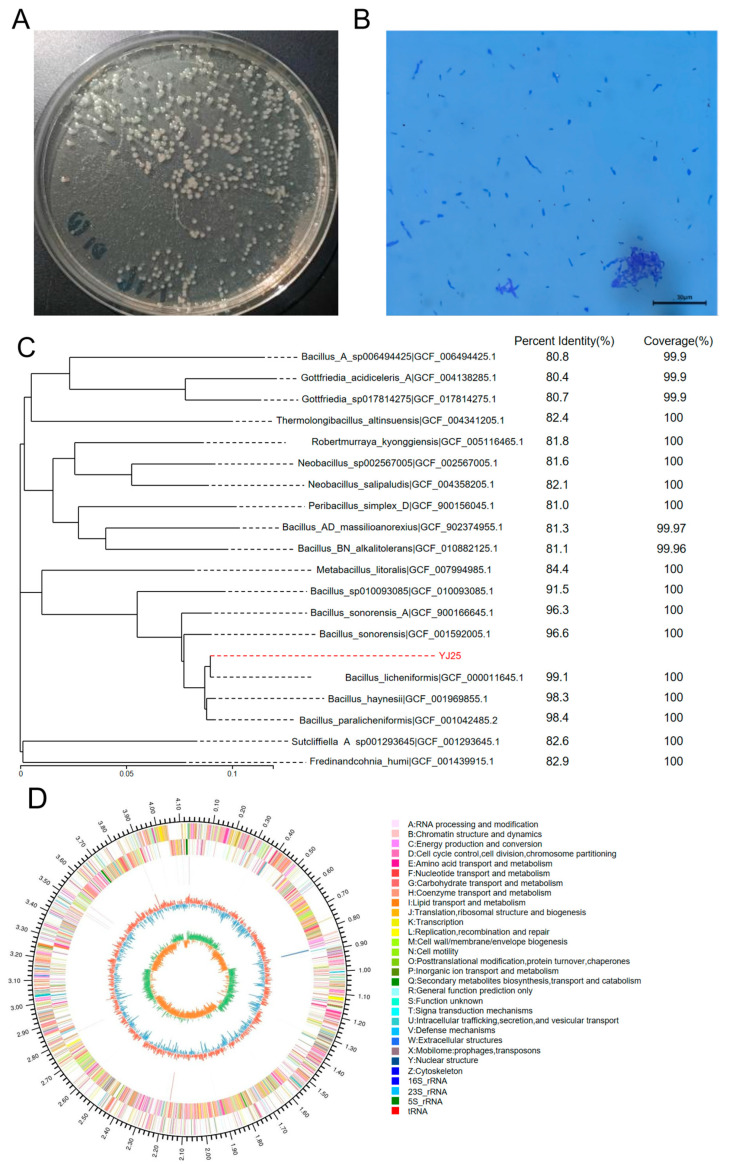
The morphological characteristics, Gram-stained image, phylogenetic tree, and circular genome map. (**A**) YJ25 grown on an LB agar plate for 10 h at 37 °C. (**B**) Gram staining results of YJ25 under a light microscope. (**C**) Phylogenetic tree based on whole genome sequencing data. (**D**) Circular genome map of YJ25. The circular map consisted of six circles. The outermost circle inward, each circle contained information about the genome: (1) Nucleotide sequence, (2) Forward CDS, (3) Reverse CDS, (4) rRNA and tRNA, (5) GC content, and (6) GC-SKEW.

When cultured on an LB agar plate, YJ25 formed round convex colonies with a yellowish to off-white coloration and a smooth mucoid surface (Figure 2A). Gram staining confirmed that YJ25 is Gram-positive, as observed under a light microscope (Figure 2B). The 16S rDNA sequence of YJ25, amplified to a length of 1272 bp, was subjected to BLAST 1.4.0 analysis against the NCBI database. The results demonstrated that YJ25 is highly homologous to *Bacillus licheniformis*, leading to the identification of YJ25 as a strain of *Bacillus licheniformis*.

Furthermore, whole genome sequencing data were used to construct a phylogenetic tree using the Neighbor-Joining (NJ) method in MEGA 6.0 software (Figure 2C). Figure 2D depicts the chromosomal ring map, illustrating the distribution of GC content, tRNAs, rRNAs, repetitive sequences, and gene functional annotations across the entire genome of *Bacillus licheniformis* YJ25. The complete genome of YJ25 spans 4,140,472 base pairs with a G+C content of 46.19%. It encompasses 4216 predicted protein-coding sequences, 79 tRNA genes, and 10 rRNA genes, highlighting the genomic complexity of this strain (Table 1).

*Bacillus licheniformis* is known to produce a wide range of degrading enzymes, including proteases and amylases [27]. Our whole genome sequencing analysis identified 63 genes encoding glycoside hydrolases and 36 genes encoding carbohydrate esterases (Table S1). These findings suggested that *Bacillus licheniformis* YJ25 may have the potential to enhance the utilization of nutrients in feed.

### 2.2. Effect of the Time Gradient on the Removal of ZEN by YJ25

**Figure 3:**
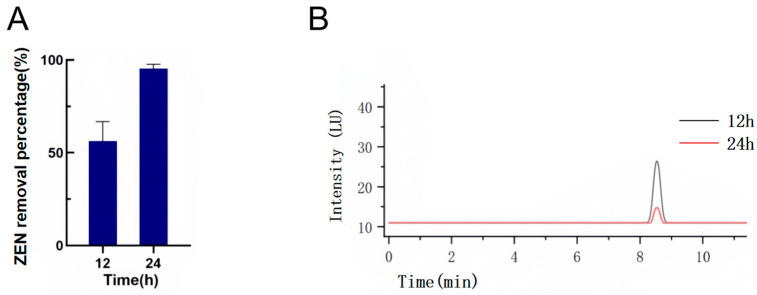
The effects of removing time on the ZEN removal ability of YJ25. (**A**) Effects of removing time on the ZEN removal ability of YJ25. (**B**) HPLC profiles of ZEN removal by different processing times of YJ25. The initial ZEN concentration was 10 μg/mL.

As reported in prior studies [23], strain CK1 demonstrated notable removal efficiency, achieving a removal percentage of 95.8% after 36 h of cultivation in LB medium containing 2 ppm ZEN. In our study, *Bacillus licheniformis* YJ25 exhibited notable removal capabilities, removing 65.27% of ZEN within 12 h. Remarkably, the YJ25 achieved a ZEN removal percentage of 95.37% within 24 h under a high concentration of 10 ppm, demonstrating comparable detoxification efficiency to CK1’s performance with 2 ppm ZEN over 36 h (Figure 3A,B). These findings suggested that *Bacillus licheniformis* YJ25 possesses enhanced ZEN removal capability and remarkable tolerance to high toxin concentrations.

### 2.3. Localization and Analysis of the Removal Active Site of YJ25

**Figure 4:**
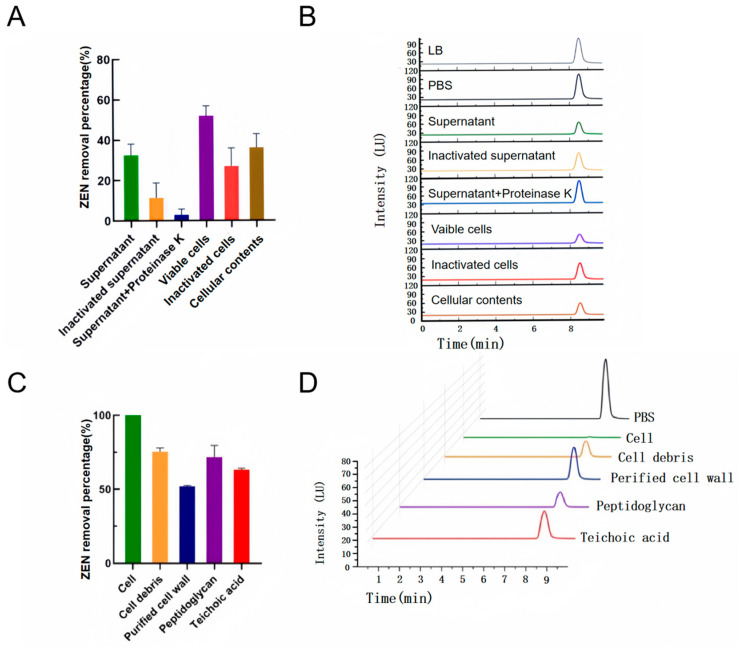
Effects of different treatments on ZEN removal by YJ25. (**A**) Removal percentage of ZEN by different cell components of YJ25. (**B**) HPLC profiles of ZEN removal by different components of YJ25. The initial ZEN concentration was 10 μg/mL. (**C**) Adsorption percentage of ZEN by bacteria prepared by different treatments. The cell removal percentage shown in the figure is 100%. (**D**) HPLC profiles of ZEN adsorption by bacteria prepared by different treatments. The initial ZEN concentration was 10 μg/mL.

After centrifugation, the supernatant was collected free of the cellular pellet. The intracellular contents were then obtained using ultrasonic cell lysis. According to Figure 4A,B, the removal percentage of ZEN by the cell-free supernatant and cellular contents was found to be 32.5% and 36.1%, respectively. These results demonstrated the presence of ZEN-removing substances in both extracellular and intracellular fractions. Interestingly, the cell supernatant’s ability to remove ZEN was markedly diminished to 8.7% after treatment with proteinase K and to 17.5% following heat treatment, indicating the presence of extracellular proteins in the supernatant, which may function as ZEN-degrading enzymes [28].

The viable cells demonstrated the highest ZEN removal percentage of 51.93%, indicating that cell wall adsorption represents the predominant mechanism of ZEN removal by YJ25. Furthermore, the adsorption capacity of cells decreased to 26.93% after heat treatment. This reduction suggested that the viable cells remained partially metabolically active during the experimental process, continuing to produce some extracellular enzymes. The reduced removal efficiency observed following heat treatment can be partly ascribed to the denaturation of extracellular enzymes but is primarily due to the structural disruption of cell wall components [29].

Our results demonstrated that YJ25’s ZEN removal is predominantly achieved through cell wall adsorption, with enzymatic degradation serving as a potential auxiliary pathway.

### 2.4. Investigation of the Cell Wall Adsorption Mechanism

The cell wall of Gram-positive bacteria is characterized by a complex structure comprising thick layers of peptidoglycan, teichoic acids, polysaccharides, and membrane proteins [30,31]. Previous studies have demonstrated that specific components of bacterial cell walls can adsorb mycotoxins. For instance, the exposed peptidoglycan layer of lactic acid bacteria has been shown to adsorb fumonisins B_1_ and B_2_ [18], while the polysaccharide component of the *Lactobacillus rhamnosus* strain can effectively adsorb ZEN [17]. However, the specific mechanism underlying ZEN adsorption by *Bacillus licheniformis* remains unexplored.

To investigate the adsorption mechanism of ZEN by *Bacillus licheniformis* YJ25, we conducted systematic treatments of YJ25 cell walls. As illustrated in Figure 4C,D, cell wall fractions exposing peptidoglycan and teichoic acid demonstrated significantly enhanced ZEN adsorption capacities compared to purified cell walls, with adsorption percentages reaching 71.75% and 63.2%, respectively. These findings demonstrated that both peptidoglycan and teichoic acids serve as critical structural components facilitating ZEN adsorption in YJ25. The observed differences in their adsorption capacities suggested distinct functional roles in the binding process, highlighting the need for further investigation into their specific interaction mechanisms with ZEN molecules.

#### FTIR Analysis Results

**Figure 5:**
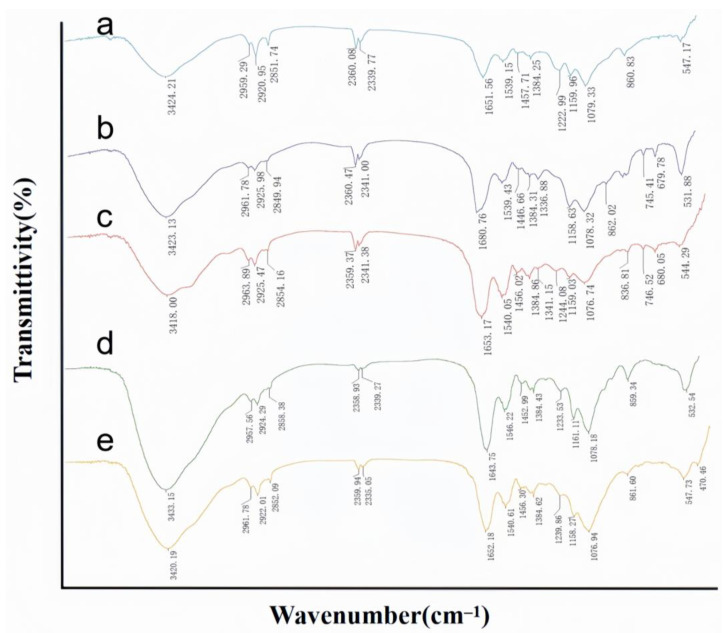
FTIR absorption spectra of bacteria prepared by different treatments. (**a**,**c**,**e**) are purified cell wall, cell wall with exposed peptidoglycan components, and cell wall with exposed teichoic acid components, respectively, before ZEN adsorption. (**b**,**d**) are cell walls with exposed peptidoglycan components and cell walls with exposed teichoic acid components, respectively, after ZEN adsorption.

FTIR is employed to identify the functional groups present on the cell surface, as each group exhibits a unique energy absorption band [32]. The detailed wave number shifts corresponding to different treatments are summarized in Table S2, while Figure 5 presents the average FTIR spectra before and after the adsorption of ZEN by cell walls exposed to peptidoglycan and teichoic acid, along with the average FTIR spectra of the purified cell walls. The FTIR spectra were analyzed according to the literature [19,33,34,35]. The absorption peaks observed at 3424.21 cm^−1^, 3418.00 cm^−1^, and 3420.19 cm^−1^ are attributed to the overlapping of broad bands resulting from the stretching of O-H and N-H bonds in proteins, sugars, and fatty acids. The peaks at 2959.29 cm^−1^, 2963.89 cm^−1^, and 2961.78 cm^−1^, as well as those at 2920.95 cm^−1^, 2925.47 cm^−1^, and 2922.01 cm^−1^, are indicative of the characteristic C-H asymmetric stretching vibrations associated with the CH_3_ and CH_2_ functional groups, which are typically found in fatty acid chains of lipids. The vibrations at 2851.74 cm^−1^, 2854.16 cm^−1^, and 2852.09 cm^−1^ correspond to the absorption of C-H bonds in proteins and lipids.

The vibrations at 1651.56 cm^−1^, 1653.17 cm^−1^, and 1652.18 cm^−1^ are ascribed to the stretching vibrations of the C=O bonds in the amide I bands of α-helical structures, which are prevalent in proteins and peptides. Similarly, the vibrations at 1539.15 cm^−1^, 1540.05 cm^−1^, and 1540.64 cm^−1^ are attributed to the N-H bending vibrations within the protein amide II bands. The peaks at 1457.71 cm^−1^, 1456.02 cm^−1^, and 1456.30 cm^−1^ are associated with C-H bending vibrations in protein peptide bonds.

Furthermore, the peaks at 1244.08 cm^−1^ and 1239.86 cm^−1^ are identified as C-N stretching vibration peaks. The vibrations at 1159.96 cm^−1^, 1159.03 cm^−1^, and 1158.27 cm^−1^ are attributed to C-O stretching vibrations in carboxylic acids. Lastly, the peaks at 1079.33 cm^−1^, 1076.74 cm^−1^, and 1076.94 cm^−1^ are attributed to the stretching vibrations of C-O and C-C bonds in sugars, which are consistent with the presence of free phosphate ion species and the lipid C-O-C bond stretching.

In summary, the FTIR analysis revealed that the cell walls with exposed peptidoglycan and teichoic acid predominantly contain O-H, N-H, C=O, C-N, and C-O bonds, which are characteristic groups of proteins, sugars, and lipids.

When comparing the purified cell walls with those exposing peptidoglycan and teichoic acid, there was a significant change in the peak shape around 1240 cm^−1^ as well as in the C-H bonding region, indicating that the treatment with trichloroacetic acid (TCA) and hydrochloric acid increased the exposure of the C-N bond to the C-H bond.

When comparing the cell walls exposing peptidoglycan and teichoic acid before and after adsorbing ZEN, the stretching vibration of the O-H bond of the strains was red-shifted by 5.13 cm^−1^ and 12.96 cm^−1^, respectively, indicating that part of hydroxyl group was involved in the adsorption, which partially broke the formed hydrogen bond and caused the hydroxyl group stretching peak to be red-shifted. The stretching vibration of the C-H bond and the bending vibration of the C-H bond of the aliphatic CH_3_ and CH_2_ were red-shifted or blue-shifted. The N-H stretching vibrations of the amide II band in the peptidoglycan-exposed cell wall underwent a red shift. Similarly, the C=O stretching vibrations of the amide I band and the N-H stretching vibrations of the amide II band in the teichoic acid-exposed cell wall also exhibited red shifts. Additionally, the C-O stretching vibrations in the carboxylic acid groups of the teichoic acid-exposed cell wall showed a red shift of 2.84 cm^−1^, which was attributed to the alteration in the carboxylic anion antagonism against the counteracting cation. Moreover, the C-O stretching vibrations of polysaccharides were all red-shifted. Earlier studies on *Bacillus licheniformis* CK1 have not explored the mechanism of its adsorption of ZEN [25]. However, a thorough understanding of this mechanism is a prerequisite for both interpreting the adsorption process and optimizing its operational efficacy in practical implementations. Our results indicated the groups involved in adsorption in the cell wall, exposing the fact that peptidoglycan and teichoic acid are mainly O-H, C-H, C=O, and C-O bonds, which suggests that polysaccharides, proteins, and lipids in the cell wall were involved in the process of adsorption.

### 2.5. Results of Degradation Product Analysis

**Figure 6:**
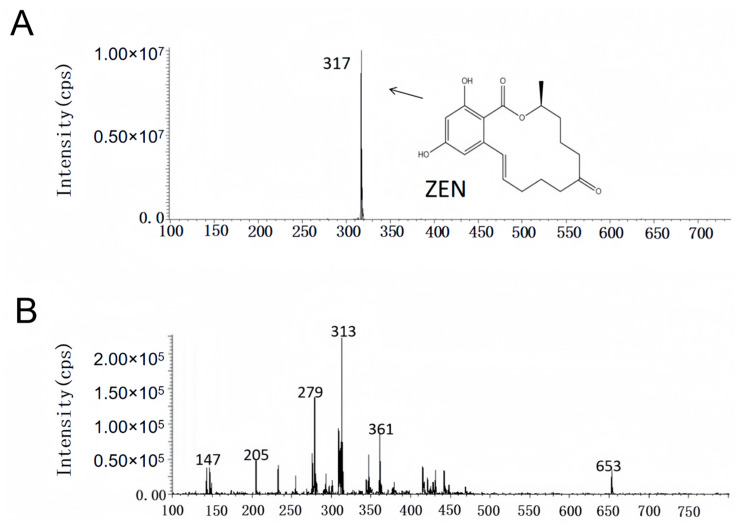
Comparison of LC–MS analysis results of degradation products and ZEN. (**A**) Positive control group (LB + 10 μg/mL ZEN). (**B**) Test group (YJ25 + 10 μg/mL ZEN).

The study of ZEN removal site analysis demonstrated that YJ25 utilizes cell wall adsorption as its dominant ZEN detoxification mechanism, while enzymatic degradation pathways may serve as an auxiliary ZEN elimination mechanism. However, bioconversion may not necessarily equate to detoxification, as the resulting metabolites may retain or even exhibit enhanced toxicity. A notable example is the enzymatic degradation of ZEN, which produces α-zearalanol and β-zearalanol—metabolites that remain estrogenic analogs with potential biological activity [15,16]. Previous studies on CK1 have not investigated whether its removal of ZEN generates secondary metabolites with higher toxicity [25]. Research on *Streptomyces rimosus* subsp. *rimosus* LMG19352 demonstrated complete degradation of 5 mg/L ZEN within 24 h; however, their results indicated that the strain first transformed ZEN into another highly estrogenic compound that acts as an intermediate product, showing delayed significant detoxification of ZEN and potential associated risks [36]. As illustrated in Figure 6A,B, the results clearly indicated the presence of a peak substance detected at approximately 10 min that was ZEN, characterized by a specific *m/z* ratio of 317 [M − H]⁻. A marked difference was observed between the test group and the positive control group, suggesting that *Bacillus licheniformis* YJ25 effectively eliminated ZEN. Notably, the characteristic peaks corresponding to α-/β-ZEL and α-/β-ZAL were not detected in the test group, substantiating the safety profile of YJ25 in the detoxification of ZEN.

## 3. Conclusions

This study identified a strain of *Bacillus licheniformis* YJ25, which exhibited a remarkable ZEN removal efficiency of 95.37% within 24 h. Experimental analysis demonstrated that the removal percentages of the cell-free supernatant and intracellular components of YJ25 were 32.5% and 36.1%, respectively, whereas the cell pellet maintained a removal percentage of 51.93%. Notably, the removal capacity of the supernatant was significantly reduced upon heat inactivation. These findings suggested that the principal mechanism of ZEN removal appears to be cell wall adsorption, with enzymatic degradation serving as a potential auxiliary pathway. The mechanistic investigations indicated that the primary adsorption sites for compounds within the cell wall are predominantly located on peptidoglycan and teichoic acid molecules. Further investigation revealed that O-H, C-H, C=O, and C-O bonds within the cell wall play a critical role in the adsorption process. Additionally, we confirmed the safety profile of YJ25 in the detoxification of ZEN. In subsequent studies, we will conduct in vivo safety assessments of YJ25, investigate its stability under varying environmental conditions, evaluate its probiotic potential using a simulated gastrointestinal model, and explore its potential adaptation over time to investigate its potential applications.

## 4. Materials and Methods

### 4.1. Sample and Primary Reagents

#### 4.1.1. Moldy Corn Sample

The moldy corn sample was purchased from Tang Ren Shen Group Co., Ltd. (Zhuzhou, China), 2024. Subsequently, the acquired corn sample was sealed in self-sealing bags and stored at 4 °C under refrigeration.

#### 4.1.2. Primary Reagents and Media

ZEN standard (purity ≥ 98%), MedChemExpress, New Jersey, USA; Chromatographic grade methanol, acetonitrile, and ethyl acetate were purchased from Sinopharm Chemical Reagent Co., Ltd., Shanghai, China; Sodium dodecyl sulfate (SDS), Sangong Biotech Co., Ltd., Shanghai, China; Proteinase K, Tiangen Biotech Co., Ltd., Beijing, China; TCA, Shanghai Macklin Biochemical Technology Co., Ltd., Shanghai, China.

LB medium (g/L): tryptone,10.0; yeast infusion powder, 5.0; sodium chloride, 10.0; pH 7.0 ± 0.1, 25 °C. Solid medium was supplemented with agar at 12.5 g/L.

### 4.2. Main Instruments

Ultra-clean bench, Tianjin Tai Sight Instrument Co., Ltd., Tianjin, China; Analytical balance, SHIMADZU, Kyoto, Japan; Vortex oscillator, Scientific Industries Inc., Delaware, USA; High-performance liquid chromatograph, Agilent, California, USA; Ultrasonic cell crusher, Ningbo Scientz Biotechnology Co., Ltd., Ningbo, China; Light microscope, Leica, Wetzlar, Germany; Benchtop mini-centrifuge, Eppendorf, Hamburg, Germany; Infrared spectrometer, Thermo Fisher Scientific Nicolet, Massachusetts, USA; Liquid chromatography-mass spectrometer, SHIMADZU, Kyoto, Japan.

### 4.3. Methods for the Determination of ZEN

The concentration of ZEN in the sample was determined by HPLC on a Welch Ultimate XB-C18 column (5 μm, 4.6 × 250 mm); mobile phase: acetonitrile: water = 60:40; flow rate: 0.6 mL/min; column temperature: 30 °C; injection volume: 20 μL; fluorescence detector: excitation wavelength (Ex) = 254 nm, emission wavelength (Em) = 440 nm.

$$ZEN\;removal\;percentage=\frac{\left(Control\;Sample\;ZEN\;Readings\;-\;Test\;Sample\;ZEN\;Readings\right)}{Control\;Sample\;ZEN\;Readings}\times 100\%$$

### 4.4. Isolation and Characterization of Efficient Removal Strains of ZEN

#### 4.4.1. Isolation of ZEN-Removal Strains

The moldy corn sample weighing 1 g was suspended in 20 mL of sterile distilled water, and 1 μL of nystatin (initial concentration: 5 μg/mL) was added. The mixture was incubated at 30 °C with shaking at 900 rpm for 2 h and then left undisturbed for 10 min. According to the protocol established by Fan Yang et al., 100 μL of the supernatant was transferred to 1 mL of LB medium supplemented with nystatin, and the initial ZEN concentration was 10 μg/mL [37]. A sterile LB medium containing 10 μg/mL ZEN served as the blank control. Each experimental group was prepared in triplicate and incubated at 30 °C with shaking at 900 rpm for 48 h, and 100 μL of the culture was transferred from each group to 1 mL of LB medium (initial ZEN concentration: 10 μg/mL) and incubated at 30 °C with shaking at 900 rpm for 48 h again. Subsequently, 100 μL of the supernatant was analyzed by HPLC to determine the ZEN concentration. If a reduction in ZEN concentration was observed, the enrichment culture process was repeated. The final enrichment culture was serially diluted and plated on an LB agar plate, followed by overnight incubation at 37 °C. Distinct colonies were selected for streaking and purification. The purified monoclonal colonies were inoculated into 1 mL of LB medium containing nystatin, supplemented with 10 μL of ZEN (initial concentration: 10 μg/mL) and cultured for 48 h at 30 °C with shaking at 900 rpm. Each treatment was performed in triplicate, with a blank control (no inoculum) included. ZEN removal percentages were determined by HPLC. The strain demonstrating the highest removal efficiency was selected for further purification through successive passages on an LB agar plate. A single colony was isolated and cultured in LB medium with shaking and preserved in 50% glycerol (*v/v*) at −80 °C, designated as YJ25.

#### 4.4.2. Identification of YJ25

(1) Morphological identification

The target strain was subjected to serial dilution and plated on an LB agar plate. Following incubation at 37 °C with continuous inversion, colony morphology was observed. Distinct colonies were selected for Gram staining, and their cellular morphology was subsequently analyzed using light microscopy.

(2) 16S rDNA identification

Monoclonal colonies were aseptically transferred to 1 mL of LB broth and cultured under shaking conditions (30 °C, 900 rpm) for 12 h. The cultures were securely sealed and submitted to Sangong Biotechnology Co., Ltd. (Shanghai, China) for sequencing analysis. The obtained sequences were subjected to BLAST analysis against the NCBI database on 11 September 2024.

(3) Whole genome sequencing analysis

Monoclonal colonies were inoculated into 1.5 mL of LB broth and incubated under shaking conditions (30 °C, 900 rpm) for 12 h. The cultures were then centrifuged at 10,000 rpm for 10 min, and the supernatant was carefully removed. The resulting pellet was transferred to 2 mL centrifuge tubes, flash-frozen in liquid nitrogen, and stored at −80 °C. The preserved samples were subsequently transferred to Meiji Bio-medical Science and Technology Ltd. (Shanghai, China) for comprehensive whole-genome sequencing analysis.

### 4.5. Effect of Time Gradient on ZEN Removal by YJ25

The influence of incubation time on the removal of ZEN by YJ25 was assessed by measuring the ZEN removal percentage at different time points. The experimental protocol was as follows: purified monoclonal colonies were inoculated into LB liquid medium and incubated at 30 °C with shaking at 900 rpm for 24 h. The culture was subsequently divided into two time-gradient groups, each comprising three replicates. Three control groups were also established. For each experimental group, 100 μL of the bacterial suspension was transferred into 1 mL of LB liquid medium containing an initial ZEN concentration of 10 μg/mL. For the control group, LB liquid medium was used instead of the bacterial suspension. All the groups were incubated at 30 °C with shaking at 900 rpm for either 12 or 24 h. The residual ZEN concentration was determined by HPLC, and the removal percentages were calculated to evaluate the impact of different incubation times on ZEN removal by YJ25.

### 4.6. Localization and Analysis of the Removal Active Site of YJ25

The strain was inoculated into 1 mL of LB medium at 30 °C with shaking at 900 rpm for 12 h. Subsequently, 200 μL of 1% inoculum was transferred to 20 mL of LB medium and incubated at 30 °C for 24 h. The cultured bacterial suspension was then centrifuged at 4 °C and 10,000 rpm for 10 min. The supernatant was collected and filtered through 0.2 μm sterile filter membrane to obtain the cell-free supernatant. The collected bacterial pellet was washed twice with sterile phosphate-buffered saline (PBS) and resuspended in an equal volume of sterile PBS to form a bacterial precipitate suspension, which was prepared into three fractions: (1) resuspended and used as the group of viable cells; (2) resuspended and inactivated at 120 °C for 20 min as a group of inactivated cells; and (3) resuspended, placed on ice, and then crushed and sonicated in an ultrasonic cell crusher. Sonication was carried out for 3 s, with 3 s intervals for 15 min until the bacterial suspension became transparent, followed by centrifugation at 10,000 rpm and 4 °C centrifugation for 10 min. The supernatant was filtered through a 0.2 μm sterile filter membrane as the cell contents group. The above components were co-cultured with ZEN (the initial concentration of ZEN was 10 μg/mL). Sterile PBS and LB medium containing the same concentration of ZEN (10 μg/mL) were used as the blank controls. Three replicates were prepared for each group and incubated at 30 °C with shaking at 900 rpm for 24 h. The residual amount of ZEN was determined by HPLC and the removal percentage was calculated.

Based on the aforementioned experiments, various inactivation treatments were applied to the cell-free supernatants: In treatment group 1, the supernatant was incubated in a water bath at 100 °C for 30 min. In treatment group 2, the cell-free supernatant was incubated in a water bath at 55 °C for 2 h in the presence of proteinase K (final concentration 1 mg/mL). The treated supernatants were then co-cultivated with ZEN at a final mass concentration of 10 μg/mL, and LB medium with the same mass concentration of ZEN (10 μg/mL) was used as a blank control. All the groups were incubated at 30 °C and 900 rpm for 24 h. The residual ZEN concentration in each treatment group was measured, and the removal percentage was calculated.

### 4.7. Investigation of the Cell Wall Adsorption Mechanism

The activated strains were cultured in LB for 12 h and then subjected to centrifugation at 8000 rpm for 5 min at 4 °C. The supernatant was removed, and the cell precipitates were washed three times with 1 mL of sterile PBS. After washing, the organisms were exposed to various chemicals, and each treatment group was repeated three times to obtain four different sets of cell wall fractions [19,38], as follows:

(1) H_2_O (100 °C,15 min), cell debris;
(2) 2% (*w*/*V*) SDS (100 °C, 15 min), purified cell wall fraction;
(3) 0.1 M HCl (100 °C, 15 min) to expose the teichoic acid portion;
(4) 10% (*w*/*V*) TCA (100 °C, 15 min) to expose the peptidoglycan fraction.

After treatment, the samples were centrifuged at 8000 rpm for 5 min, the supernatant was discarded, and the bacteria were resuspended after washing the precipitate twice with 1 mL PBS.

ZEN working solution was added to the above bacterial cell wall treatment groups to achieve a final concentration of 10 μg/mL, and the mixtures were incubated at 30 °C with shaking at 900 rpm for 2 h. The live cell group and the non-treated group were used as controls.

Based on the aforementioned procedures, infrared spectroscopic analyses were conducted. After exposing the components, the bacterial samples were placed in a −80 °C freezer for overnight freezing, followed by lyophilization in a freeze-dryer until the bacteria were in a powdered form. Subsequently, 200 mg of KBr was mixed with 2 mg of powdered bacteria, and the mixture was pressed and dried into pellets. FTIR was employed for the analysis, and the data were processed using OMNIC software 9.2.86. The functional groups present in the samples were identified based on the degree of change in the characteristic absorption peaks within the spectra.

### 4.8. Analysis of the Degradation Products of YJ25

ZEN was added to the activated bacterial liquid to achieve a final concentration of 10 μg/mL, and the mixture was treated at 30 °C with shaking at 900 rpm for 12 h. Sample pre-treatment was performed according to the previous steps, and the samples with detoxification effect were diluted by factors of 10, 100, and 1000. Subsequently, the metabolites were further detected by mass spectrometry.

Mass spectrometry conditions: heated electrospray ionization source temperature of 300 °C; capillary voltage of 3.2 kV; ion transfer tube temperature of 320 °C; sheath gas of 35 unit, auxiliary of 10 unit. full scan/ddms2 scanning mode: the acquisition range of 200–800 Da; the resolution of the first stage of mass spectrometry is 70, 000 FWHM, and the resolution of the second stage is 17, 500 FWHM; column: Welch Ultimate XB-C18 (5 μm, 4.6 × 250 mm); column temperature: 30 °C; injection volume: 20 μL; mobile phase: acetonitrile: water=60:40; flow rate: 0.6 mL/min.

## Figures and Tables

**Table 1:** The general features of the *Bacillus licheniformis* YJ25 genome.

Feature	Value
Genome size (bp)	4,140,472
G+C content (%)	46.19
Protein-coding genes (CDSs)	4216
rRNA genes	10
tRNA genes	79

## Data Availability

The original contributions presented in this study are included in the article/Supplementary Materials. Further inquiries can be directed to the corresponding authors.

## Supplementary Materials

The following supporting information can be downloaded at https://www.mdpi.com/article/10.3390/toxins17060263/s1, Table S1: The number of carbohydrate-active enzyme (CAZyme) genes in YJ25; Table S2. FTIR bands observed for the various treated cell walls before and after ZEN adsorption.

## Abbreviations

The following abbreviations are used in this manuscript:

ZEN	Zearalenone
LC–MS	Liquid Chromatography–Mass Spectrometry
FTIR	Fourier-Transform Infrared Spectroscopy
AF	Aflatoxins
FUM	Fumonisin
OTA	Ochratoxin A
DON	Deoxynivalenol
EU	European Union
Hbl	Hemolysin BL
Nhe	Non-hemolytic enterotoxin
HPLC	High Performance Liquid Chromatography
TCA	Trichloroacetic Acid
SDS	Sodium Dodecyl Sulfate
PBS	Phosphate-Buffered Saline
