# Supplementary material for: Isolation and Mechanistic Investigation of the Efficient Zearalenone-Removing Strain Bacillus licheniformis YJ25
Source: Toxins (Basel). 2025 May 23;17(6):263. doi: 10.3390/toxins17060263 (PMC12197482; doi:10.3390/toxins17060263)
Supplement: Supplementary file 1 [file toxins-17-00263-s001.zip › toxins-3586042-supplementary.pdf]

Supplementary Materials: Isolation and Mechanistic Investigation of the Efficient Zearalenone-Removing Strain Bacillus licheniformis YJ25

Yuting Wu [1,†], Feina Wu [1,†], Pan Zhao [1,†], Yan Gao [1], Mengyao Li [1], Mengjiao Luo [1], Qian Zhou [1], Siyuan Zhou [1], Xinhui Li [1], Yaling Hong [1], Yang Wu [1], Zhaorong Zhou [1], Yang Liu [1], Yandong Xia [2,*], Lijun Zou [3,*] and Jia Yin [1,*]

Table S1. The number of carbohydrate-active enzyme (CAZyme) genes in YJ25

Class Denfinition	Genes Number
Auxiliary Activities	7
Carbohydrate-Binding Modules	3
Carbohydrate Esterases	36
Glycoside Hydrolases	63
Glycosyl Transferases	37
Polysaccharide Lyases	10

Table S2. FTIR bands observed for the various treated cell walls before and after ZEN adsorption.

Functional group	Wavenumber/cm^{-1}		
	Perified cell wall	10% Trichloroacetic acid-treated YJ25	0.1M HCl-treated YJ25
O-H/N-H Stretching	3424.21	3418.00(3423.13)	3420.19(3433.15)
C-H Stretching（CH_3）	2959.29	2963.89(2961.78)	2961.78(2957.56)
C-H Stretching（CH_2）	2920.95	2925.47(2925.98)	2922.01(2924.29)
C-H stretching (Fatty acids)	2851.74	2854.16(2849.94)	2852.09(2858.38)
C=O stretching AmideI	1651.56	1653.17（1680.76）	1652.18（1643.75)
N-/H stretching AmideII	1539.15	1540.05(1539.43)	1540.64(1546.22)
C-H Bending	1457.71	1456.02(1446.66)	1456.30(1452.99)
C-N Stretching	*	1244.08(*)	1239.86(1233.53)
C-O stretching （Carboxylic acids）	1159.96	1159.03(1158.63)	1158.27(1161.11)
C-O and C-C stretching (Sugars)	1079.33	1076.74(1078.32)	1076.94(1078.18)
C-H out-plane bending	860.83	836.81(862.02)	861.60(859.34)
	*	746.52(745.41)	*(*)
	*	680.05(679.78)	*(*)
	547.17	544.29(531.88)	547.73(532.54)
	*	*(*)	*(470.46)

The wave numbers obtained from cell walls after ZEN adsorption are shown in parentheses.
